# Primary Prevention of Pediatric Asthma through Nutritional Interventions

**DOI:** 10.3390/nu14040754

**Published:** 2022-02-10

**Authors:** Giulia Nuzzi, Maria Di Cicco, Irene Trambusti, Massimo Agosti, Diego G. Peroni, Pasquale Comberiati

**Affiliations:** 1 Department of Clinical and Experimental Medicine, Section of Pediatrics, University of Pisa, 56126 Pisa, Italy; giulianuzzi92@gmail.com (G.N.); mariaelisa.dicicco@gmail.com (M.D.C.); irene.trambusti@yahoo.it (I.T.); pasquale.comberiati@unipi.it (P.C.); 2 Woman and Child Department, Division of Neonatology and Pediatrics, “F. Del Ponte” Hospital, University of Insubria, 21100 Varese, Italy; massimo.agosti@uninsubria.it; 3 Department of Clinical Immunology and Allergology, I.M. Sechenov First Moscow State Medical University, 119991 Moscow, Russia

**Keywords:** asthma, breastfeeding, children, complementary feeding, maternal diet, omega-3 fatty acids, prevention, vitamin D, wheezing

## Abstract

Asthma is the most common chronic non-communicable disease in children, the pathogenesis of which involves several factors. The increasing burden of asthma worldwide has emphasized the need to identify the modifiable factors associated with the development of the disease. Recent research has focused on the relationship between dietary factors during the first 1000 days of life (including pregnancy)—when the immune system is particularly vulnerable to exogenous interferences—and allergic outcomes in children. Specific nutrients have been analyzed as potential targets for the prevention of childhood wheeze and asthma. Recent randomized controlled trials show that vitamin D supplementation during pregnancy, using higher doses than currently recommended, may be protective against early childhood wheezing but not school-age asthma. Omega-3 fatty acid supplementation during pregnancy and infancy may be associated with a reduced risk of childhood wheeze, although the evidence is conflicting. Data from observational studies suggest that some dietary patterns during pregnancy and infancy might also influence the risk of childhood asthma. However, the quality of the available evidence is insufficient to allow recommendations regarding dietary changes for the prevention of pediatric asthma. This review outlines the available high-quality evidence on the role of prenatal and perinatal nutritional interventions for the primary prevention of asthma in children and attempts to address unmet areas for future research in pediatric asthma prevention.

## 1. Introduction

Asthma is the most common chronic non-communicable disease of the pediatric age, affecting 5–10% of school-aged children and adolescents [1]. It is a heterogeneous disease that results from the interaction between several genetic, epigenetic, and environmental factors. The increasing burden of asthma worldwide has emphasized the need to identify the modifiable factors associated with its development [1].

Recent research has highlighted the role of exogenous environmental exposures during prenatal and early postnatal life in the pathogenesis of asthma. These factors include tobacco smoke, air pollutants, indoor and outdoor aeroallergens, respiratory infections, and loss of biodiversity [2,3].

Nutrition in the first 1000 days of life, defined as the period between conception and the first 2 years of life, is also emerging as an important driver of long-term respiratory health [4,5]. Recent evidence points toward some dietary factors, including maternal and infant diet composition, nutrients status, in particular vitamin D (VD) and omega-3 fatty acids (Table 1 and Table 2), breastfeeding, the use of hydrolyzed milk formulas, and the time of introduction of complementary foods, as potential targets for the prevention of childhood-onset asthma [2]. However, current evidence provides limited information on dietary interventions during pregnancy, lactation, and infancy to prevent asthma, as acknowledged in the recent Global Initiative for Asthma (GINA) guidelines [1].

This review outlines the available high-quality evidence on the role of nutritional interventions during prenatal and perinatal life for the primary prevention of pediatric asthma and attempts to address unmet areas for future research in this field.

## 2. Methods

A literature search was performed in September 2021 across MEDLINE/PUBMED to identify studies investigating nutritional interventions during prenatal and early life associated with the primary prevention of asthma in children. We included randomized controlled trials (RCTs), observational (cross-sectional and cohort), and case-control studies, which (a) considered either pregnancy or childhood, (b) reported asthma/wheezing in the offspring, (c) were peer-reviewed, and (d) were written in English. Both the general population and populations at high risk of atopy were included. The search criteria were diet* OR Mediterranean diet* OR nutrient* OR nutrition* OR food* OR fatty acid* OR Omega-3* OR Omega-6* OR PUFA* OR Dairy product* OR breastfeeding* OR breast milk* OR infant formula* OR hydrolyzed formula* OR vitamin* OR probiotic* OR prebiotic* OR *pregnancy* OR infancy* OR maternal diet* OR infant diet* AND prevention* AND asthma* OR wheeze* AND preschool children* OR infant*. The special interest was in studies published within the previous 24 months.

## 3. Pregnancy and Lactation

### 3.1. Maternal Dietary Patterns

Diet constitutes an essential source of nutrients and non-nutrient components, which, during the critical time frame of pregnancy and lactation, can influence the immune system development of the newborn, shape the composition of the human microbiome and influence gene expression, thereby potentially affecting the risk of asthma in children [25,26]. In this regard, a recent European cohort study conducted on lactating women showed that various dietary patterns do not adequately supply all nutrients, indicating the need for further promoting overall healthy dietary habits in this population [27].

A recent prospective study showed that maternal consumption of specific foods during pregnancy, such as cooked green vegetables, may protect against childhood asthma, while a high intake of meat during the preconception period may increase the risk of wheezing, allergic rhinitis, and atopic dermatitis in children by the age of 3 years [28]. Another recent birth cohort study showed that a healthy diet during pregnancy, as measured by the maternal diet index, was associated with up to 20% reduced likelihood of wheeze and 16% reduced likelihood of asthma in offspring up to age four [29].

Adherence to a Mediterranean diet during pregnancy is often reported as a protective factor against the development of allergy and asthma [30,31], but evidence regarding childhood asthma is conflicting [32]. In an observational study, Castro-Rodriguez et al. reported that low consumption of potatoes and pasta by mothers during pregnancy was associated with a lower risk of wheezing in preschool children, but maternal adherence to the Mediterranean diet was not a protective factor for this outcome [33].

Moreover, information about the effect of the Western diet and fast food consumption during pregnancy on the risk of pediatric asthma is conflicting. A recent cohort study suggested that a proinflammatory and low-quality diet during pregnancy is associated with a higher risk of offspring asthma in the first 10 years of life [34]. Hanson et al. found an association between the maternal diet inflammatory index and wheezing trajectories during early childhood but not asthma [35]. In a recent prospective study, Venter et al. found that elevated scores on the maternal dietary inflammatory index slightly increased the risk of offspring wheezing at the age of 4 years. While it might be of clinical relevance, this finding was not statistically significant in an adjusted model [36]. Data from the same cohort study showed that maternal intake during pregnancy of advanced glycation end products (AGEs), which are contained in foods typical of the Western diet, such as burgers and fried foods, was not associated with offspring asthma and allergy outcomes up to age eight [37]. Other observational studies found either no significant association between maternal diet quality during pregnancy and asthma at 10 years old [38] or even a protective effect of a maternal Western diet pattern in pregnancy on the risk of childhood wheezing [39].

A recent systematic review of observational studies concluded that intake of foods associated with a Mediterranean diet, fish/fatty fish, tree nuts, fruit, vegetables, meat (type of meat not specified), vitamin D (VD), and zinc during pregnancy was associated with a reduced risk of asthma or wheeze in children. However, the doses of foods or nutrients, as well as the methods used for measuring dietary intake, were heterogeneous between studies, which limits the ability to provide clear practical recommendations at this time [31].

Given the complexities of studying the effect of maternal diet during pregnancy and lactation on the risk of asthma in children, there is a need for confirmatory RCTs, which would need to investigate beyond single nutrients/foods, consider nutrient intake/levels before the intervention, and possibly have a follow-up at school age, as not all children who wheeze in the first few years of life will later develop asthma.

### 3.2. Maternal Intake of Vitamins

VD, vitamin A, and vitamin E have known immunomodulatory activities [40]. In addition, there is evidence that VD contributes to lung growth during the fetal–neonatal life and modulates both innate and adaptive immunity, inhibiting some proinflammatory responses associated with allergy and asthma [41]. Prospective and cross-sectional studies have reported an inverse association between maternal VD status and the risk of asthma in the offspring [41]. Recent evidence from RCTs supports a protective role for VD sufficiency throughout pregnancy, particularly in reducing the risk conferred by maternal asthma on childhood recurrent wheeze or asthma [42].

Table 1 summarizes the findings of RCTs on VD supplementation during pregnancy and lactation with offspring asthma/wheeze as an outcome [6,7,8,9,10,11,43]. In the Vitamin D Antenatal Asthma Reduction Trial (VDAART), 881 women with a high risk of atopy were randomized to receive either high (4400 IU) or standard (400 IU) VD3 daily supplementation during the last 2 semesters of gestation [6]. The children born from these women showed a clinically relevant, but not statistically significant, reduction (20% or greater) in the risk of asthma/recurrent wheezing by the age of 3 years. Similar findings were reported by the COPSAC study [7,9], which enrolled women from an unselected cohort and used high (2400 IU) versus standard (400 IU) VD3 daily supplementation during the third trimester of gestation [8,43]. However, the six-year follow-up analysis of both the VDAART and COPSAC study showed that high dose VD supplementation during pregnancy did not affect the risk of asthma by the age of 6 years [7,9,10]. These findings suggest that increasing VD supplementation during pregnancy may be effective in preventing transient preschool wheezing, which is most commonly viral-induced (potentially due to the immunomodulating and antiviral effects of VD), but not school-age asthma, which is a heterogeneous disease most often associated with allergic sensitization to aeroallergens [44].

Regarding the potential role of supplementation with other types of vitamins, there are only a few RCTs that report conflicting results [45,46,47]. Overall, the current evidence on vitamin supplementation during pregnancy to prevent childhood asthma is heterogeneous [31]. Further studies are needed before supplementation with high doses of VD during pregnancy could be recommended as a strategy to prevent childhood wheeze.

### 3.3. Maternal Intake of Omega-3 Fatty Acids

There is increasing evidence to suggest that omega-3 long-chain polyunsaturated fatty acids (LCPUFAs) have antiinflammatory properties and may modulate the risk of allergic disease [31]. Fish and fish oil are significant sources of omega-3 LCPUFAs and research that investigated the effect of maternal fish oil supplementation during pregnancy showed a reduced risk of allergic sensitization to aeroallergens in the offspring [13,16,48,49]. However, other authors found no difference in the incidence of allergic disease [50]. A recent Cochrane review found that there is limited evidence to support that supplementation with LCPUFAs during pregnancy and lactation may reduce the incidence of allergy in children [51], and even concerning the association between LCPUFA supplementation during pregnancy and decreased incidence of wheezing in preschoolers, the data show mixed results [12,13,14,15,16,52] (Table 1). A recent meta-analysis concluded that omega-3 LCPUFA supplementation during pregnancy is not associated with a significant protective effect on wheezing or asthma in the offspring [53]. The most recent systematic review and meta-analysis of RCTs showed that maternal supplementation/intake during pregnancy with omega-3 LCPUFAs was associated with a trend toward a protective effect on wheeze/asthma outcomes in the offspring (OR: 0.70; 95% CI: 0.45–1.08), but this finding did not reach statistical significance [31]. The latest position paper from the European Academy of Allergy and Clinical Immunology (EAACI) on the influence of dietary fatty acids on allergy concluded that although such nutrients may have immunomodulatory properties, the lack of standardized formats (i.e., food versus supplement) and doses, as well as the lack in many clinical studies of serum fatty acid level assessments before the intervention, significantly limit the possibility to compare allergy outcomes across studies and provide clear recommendations [52].

### 3.4. Maternal Intake of Prebiotics and Probiotics

There is increasing evidence to show the relevant role of the human microbiome in modulating the maturation and function of the immunological system [54]. Thus, oral supplementation of probiotics and prebiotics in the prenatal period has been investigated in recent decades as a possible strategy to shape the microbiome composition towards the development of tolerogenic immune responses. However, the evidence on asthma and allergy outcomes is scarce. Prebiotics are defined as ‘dietary ingredients, selectively fermented, that can cause specific changes in the gut microbiota’s composition and/or activity, thus conferring health benefits to the host’ [55]. In contrast, probiotics can be defined as dietary supplements that contain live microbial strains capable of persisting in (or transiently colonizing) the human intestinal tract [56]. However, there is a lack of data on prebiotic supplementation during pregnancy/lactation to prevent asthma in the offspring [57]. Different meta-analyses showed that maternal supplementation with probiotics during pregnancy or lactation did not reduce the risk of wheeze or asthma in the offspring [58,59,60]. Currently, available data do not support the use of prebiotics and probiotics during prenatal life for the prevention of wheeze and asthma in infants.

## 4. Breastfeeding

Breast milk comprises a wide range of immunological nutrients and bioactive components, such as LCPUFAs, oligosaccharides, proteins, free amino acids, immunoglobulin A, antimicrobial peptides, vitamins, and cytokines, which are important for the development and maturation of the infants’ innate and adaptive immune system [61]. However, whether breast milk and breastfeeding duration protect against the development of allergy and asthma is controversial [62].

Previous systematic reviews have reported a protective effect of breastfeeding on allergic outcomes, although many of the included studies have methodological limitations [63]. A systematic review by Dogaru et al. [64] showed that children who were breastfed longer had a lower risk of developing childhood wheezing and asthma. Risk reduction was more significant in children 0–2 years of age and diminished over time. This finding would suggest that breastfeeding may protect against transient virus-induced preschool wheezing. On the other hand, this protection tends to wane in older children when heterogeneous factors can influence respiratory morbidity and persistent asthma, which is usually an allergic-type of asthma. However, the included studies were highly heterogeneous [63]. The latest systematic review and meta-analysis by the UK Food Standards Agency did not find any evidence for the duration of breastfeeding on asthma prevention in the offspring [62].

Although a clear association between breastfeeding and asthma prevention has not been established, breastfeeding has multiple health benefits for infants and mothers and should be encouraged wherever possible [65,66].

## 5. Infancy

### 5.1. Use of Hydrolyzed Infant Formulas

Over the past decade, hydrolyzed milk formulas (HF), which include partially hydrolyzed formula (pHF) and extensively hydrolyzed formula (eHF), have been recommended in many countries for the potential prevention of allergic diseases in infants at high risk of atopy who cannot be breastfed [67,68]. However, a recent meta-analysis does not support this recommendation, especially regarding asthma prevention. A recent study by Davisse-Paturet et al. [69], analyzed the association between childhood asthma and either the use of breast milk only or pHF or non-hydrolyzed milk formula. These researchers showed that the use of pHF compared to non-hydrolyzed formula had no protective effect on asthma risk up to 2 years of age and was linked to a higher risk of wheezing at 1 year in infants at high risk of atopy. Moreover, a 2018 Cochrane review found no evidence to support short-term or prolonged use of either pHF or eHF, compared with exclusive breastfeeding, to prevent allergic disease including childhood asthma. It is of note that the quality of evidence was very low for all allergic outcomes [70].

There is a need for further evidence, using robust study designs, about the impact on childhood wheeze and asthma prevention of hydrolyzed formulas currently on the market versus conventional cow’s milk non-hydrolyzed formula in the first months of life.

### 5.2. Timing of Introduction of Solid Foods

Recent evidence on the primary prevention of allergic diseases shows that the early 2000s recommendation to delay the introduction of solid allergenic foods to the infant’s diet until 1 year of age or later is not a protective intervention against the development of atopic outcomes in children [71]. Observational studies report conflicting evidence regarding the association between early complementary feeding and the prevention of childhood asthma, with some studies reporting that the early introduction (before 1 year of age) of some solids, such as fish and cereals, is associated with a reduced risk of wheeze or asthma in children [72,73]. In contrast, others suggest that the nature and timing of complementary feeding do not substantially influence the long-term risk of pediatric asthma [74].

The timing of the introduction of fish is of particular interest to the purpose of the prevention of pediatric asthma given its high content in omega-3 fatty acids. A recent meta-analysis of observational studies concluded that introducing fish early in life (6–9 months) and regular consumption of all fish (at least once a week) reduces asthma and wheeze in children up to 4.5 years old [75]. Another meta-analysis, which included both intervention and observational trials, concluded that there is low-to-very low certainty evidence that early fish introduction is associated with a reduced a risk of rhinitis and allergic sensitization. In contrast, there is conflicting evidence regarding the prevention of childhood wheezing [76].

A recent high-quality clinical trial showed that the early introduction of peanuts in high-risk infants significantly reduced the risk of peanut allergy but had no preventive effect on the development of asthma and respiratory allergy [77].

### 5.3. Dietary Patterns

As people consume different food groups in a meal, the analysis of different dietary patterns versus single foods or small food groups is more appropriate for understanding the effect of nutrition on the risk of developing asthma [78].

There is observational evidence that Mediterranean diet exposure, assessed through the Mediterranean diet index, is associated with a lower prevalence of asthma in children [79]. One possible explanation for the observed beneficial effects is that the Mediterranean diet is characterized by low consumption of red meat and saturated fat, and a high intake of fruits, vegetables, whole grains, legumes, fish, and olive oil, which are rich in antioxidants, micronutrients, and VD. These compounds limit the inflammatory response in the airways, thereby possibly reducing the risk of asthma [80]. Conversely, the Western dietary pattern, characterized by increased consumption of foods rich in AGEs, i.e., meats and saturated fats [81], may promote activation of the toll-like receptor pathway and NF-κB inflammatory cascade, thereby contributing to airway inflammation and asthma pathogenesis [82]. Indeed, higher meat consumption and increased AGE intake are associated with childhood wheezing [83,84]. A recent cohort study demonstrated that a proinflammatory diet is associated with increased wheezing in atopic children [85]. These findings support the epidemiological association between fast food consumption and the increasing prevalence of asthma [86] but are in contrast with the results of the Dutch Generation R cohort study, where an association between a healthy dietary pattern in early life and a lower risk of childhood asthma was not found [38].

Food diversity in the first year of life may also contribute to the risk of allergic outcomes in children. Prospective cohort studies reported that an increased food diversity within the first year of life reduced the risk of asthma by the age of 6 years [87]. In contrast, a reduced diet diversity at the age of 1 year increased the risk of childhood wheeze and asthma at age five [88]. Further research is required to investigate the relationship between diet quality and diversity and the prevalence of asthma, and whether food intake patterns in early life are associated with later allergy and asthma development.

### 5.4. Intake of Vitamins

Epidemiological evidence suggests that VD deficiency is associated with the prevalence of childhood wheeze and asthma [89,90]. Low VD serum levels are associated with increased type-2 responses, interleukin-10 production, and reduced T-regulatory cells activity [91]. Early postnatal colonization of the airways by pathogenic bacteria is influenced by VD status and represents a risk factor for asthma development [92]. Emerging data suggest that VD can improve the antiinflammatory response of corticosteroid therapy and potentially be used as an adjuvant therapy in steroid-resistant asthma [93].

Observational studies have often demonstrated a correlation between low VD levels in early life and the development of wheezing in preschool and asthma later in life [94,95]. In an Australian cohort study conducted in a high-risk population, VD deficiency in early childhood was associated with a higher risk of persistent asthma at 10 years of age and an increased risk of early allergic sensitization and susceptibility to respiratory infections [96], which are known to be risk factors for wheezing and asthma [97,98].

However, supplementation trials have reported mixed results. Among atopic infants who had sufficient VD status at birth, supplementation with 400 UI VD3 daily for the first 6 months of life did not modify the risk of wheeze at 1 year or of recurrent wheeze or asthma at 2.5 years compared to a placebo [17]. On the contrary, supplementation with 400 UI VD3 daily for the first 6 months of life resulted in protection from wheeze or asthma in early infancy when the intervention was applied to either premature black infants [18] or both mothers during pregnancy and infants [19]. Of note, the results from both in utero and postnatal VD supplementation trials suggest that VD may reduce susceptibility to transient viral-induced preschool wheezing (Table 1 and Table 2). However, there are insufficient data to address the role of VD supplementation in infants for the prevention of persistent school-age asthma [99]. Further intervention trials with long-term follow-up are needed to evaluate the combined effect of prenatal and postnatal VD supplementation in the primary prevention of asthma. In addition, future studies would have to consider the role of sun exposure and lifestyle changes as a means to correct VD levels because solar ultraviolet radiation of the skin provides 90–95% of the total VD requirements.

### 5.5. Intake of Omega-3 Fatty Acids

The administration of omega-3 LCPUFAs in early life has been proposed to prevent allergic diseases, including asthma [100,101], as LCPUFAs have been shown to modulate immune and inflammatory responses [102]. However, conflicting results have been reported in studies evaluating the supplementation of omega-3 LCPUFAs, through the administration of fish oil, to prevent recurrent wheeze or asthma in early childhood [20,21,22,23,24,103,104,105] (Table 2). Of note is that elevated plasma levels of omega-3 fatty acids [20,24] and maternal history of allergy [22] were associated with a reduced risk of recurrent wheeze in those trials reporting a protective effect of LCPUFA supplementation. Overall, the available studies show methodological heterogeneity and the risk of suboptimal adherence bias. Further clinical trials with long-term follow-up are needed to clarify the role of LCPUFA supplementation during the first years of life in the prevention of pediatric asthma.

### 5.6. Intake of Prebiotics and Probiotics

There is accumulating evidence to show that the gut and lung microbiome maturation and composition in early life can affect the long-term risk of allergic diseases, including asthma [106]. A recent prospective study has shown that bacterial maturity and diversity of the gut microbiome in the first months of life influence the risk of asthma at school age, especially in children born to asthmatic mothers [107]. Over the last decade, researchers have tried to modulate the gut microbiome composition, through the administration of oral probiotics and prebiotics, as a means to prevent chronic diseases. Again, studies having asthma as an outcome have reported mixed results [106]. Peldan et al. [108] analyzed children at high risk of asthma showing that the prevalence of asthma at 5 and 10 years of age in the group receiving a mixture of oral probiotics for the first 6 months of life was similar to the placebo group. A recent RCT using oral supplementation of *Lactobacillus rhamnosus GG* during the first 6 months of life failed to show any significant difference in asthma at 5 years of age in high-risk infants [109]. None of the published systematic reviews and meta-analyses confirmed the protective effect of probiotic administration in the first years of life on the risk of childhood wheezing and asthma [58,59,60]. The latest meta-analysis on the topic reported that supplementation with probiotics during early life reduced the incidence of wheeze only in a small subgroup of atopic infants. However, this finding should be interpreted with caution since it was derived from a pooled subgroup analysis of a small sample size [60].

Similar to probiotics, the effect of prebiotic administration on the risk of pediatric asthma is controversial. Results from a recent RCT involving 132 infants at risk of atopy followed at the 2-year follow-up showed that infants who were fed with a formula containing a mixture of prebiotic oligosaccharides reported a lower incidence of recurrent wheezing than the placebo group [110]. A recent meta-analysis of RCTs concluded that prebiotics decreased the risk of wheeze or asthma when compared to the control group, but this finding should be interpreted with caution due to the small number of patients and events, as well as inconsistencies in defining wheeze and asthma [57]. In contrast, a Cochrane systematic review reported no association between prebiotic supplementation and childhood asthma prevention [111].

### 5.7. Intake of Unpasteurized Milk

Although raw cow’s milk consumption is not recommended due to the potential risk of pathogen contamination, there is observational evidence to show that the consumption of unpasteurized milk in early life is inversely related to childhood asthma, whereas heating the milk reduces such association [112,113]. The high content of PUFAs, bacteria, proteins, and vitamins in raw cow’s milk may confer antiinflammatory properties and explain the relationship with the risk of asthma [114]. The Milk Against Respiratory Tract Infections and Asthma (MARTHA) trial is currently testing the protective effect of microbiologically safe, minimally processed cow’s milk against standard ultra-heat-treated milk in early infancy for the primary prevention of childhood asthma [115].

## 6. Conclusions

The last few decades have seen a significant increase in the prevalence of asthma and allergic diseases, prompting global attention to identify modifiable risk factors susceptible to prevention strategies. Nutritional and dietary factors are emerging as important determinants of asthma risk in children. Recent RCTs showed that VD supplementation during pregnancy, using higher doses than currently recommended, may be protective against early childhood wheezing but not school-age asthma. Omega-3 fatty acid supplementation during pregnancy and infancy may be associated with a reduced risk of childhood wheeze, although the evidence is conflicting. Data from observational studies suggest that some dietary patterns during pregnancy and infancy might also influence the risk of childhood asthma. However, the quality of the available evidence is insufficient to allow recommendations regarding dietary changes for the prevention of pediatric asthma. Further high-quality research, in terms of study design, type and duration of interventions, and outcome measures, is needed to accurately identify modifiable nutritional risk factors for asthma and to understand whether modulation of these factors could contribute to the primary prevention of pediatric asthma.

## Figures and Tables

**Table 1:** Randomized controlled trials on vitamin D or omega-3 fatty acid supplementation during pregnancy/lactation for the primary prevention of pediatric wheeze/asthma.

Authors, Years	Population (*N*), Characteristics	Time of Exposure	Interventions	Outcomes	Findings on Wheeze/Asthma
Litonjua 2016 [6]	876 pregnant women, high-risk cohort for asthma	Pregnancy	High dose (4400 IU VD3/day) vs. standard dose (400 IU VD3/day) VD supplementation, starting at 10–18 weeks of gestation until delivery	Asthma or recurrent wheezing in offspring at 3 years of age	No statistically significant reduced risk of persistent wheeze; however, a clinically important protective effect could not be excluded (hazard ratio, 0.8; 95% CI, 0.6–1.0; *p* = 0.051)
Litonjua 2020 [7]				Asthma or recurrent wheezing in offspring at 6 years of age	No effect on the incidence of asthma and recurrent wheeze at age 6 years
Chawes 2016 [8]	623 pregnant women, unselected cohort	Pregnancy	High dose (2400 IU VD3/day) vs. standard dose (400 IU VD3/day) VD supplementation, starting at 24 weeks of gestation until delivery	Persistent wheeze and asthma in offspring at 3 years of age	No statistically significant reduced risk of persistent wheeze; however, a clinically important protective effect could not be excluded (hazard ratio, 0.76 [95% CI, 0.52–1.12]; *p* = 0.16)
Brustad 2020 [9]				Asthma in offspring at 6 years of age	No effect on child’s risk of asthma by the age of 6 years
Goldring 2013 [10]	180 pregnant women	Pregnancy	No VD vs. 800 IU VD2 daily from 27 weeks gestation until delivery vs. single oral bolus of 200,000 IU VD3 at week 27 of gestation	Wheezing illnesses (assessed by validated questionnaire) in offspring at 3 years of age	No effect on the risk of wheezing (risk ratio 0.86; 95% CI 0.49, 1.50; *p* = 0.69)
Norizoe 2014 [11]	164 mothers of infants with facial eczema at 1 month of age	Lactation	800 IU VD3 vs. placebo daily, for 6 weeks	Infantile eczema at the 3-month check-up (primary outcome). Atopic dermatitis, food allergy, and wheeze diagnosed by doctors up to 2 years of age, assessed by questionnaire (secondary outcomes).	No effect on child’s risk of wheeze (risk difference 0.11; 95% CI −0.05, 0.26; *p* = 0.19)
Olsen 2008 [12]	533 pregnant women, unselected cohort	Pregnancy	Capsule with fish oil (2.7 g n-3 PUFAs) vs. capsules with olive oil vs. no oil capsules, daily from 30 weeks of gestation until delivery	Asthma at 16 years of age	The hazard rate of asthma was reduced by 63% (95% CI: 8%, 85%; *p* = 0.03), whereas the hazard rate of allergic asthma was reduced by 87% (95% CI: 40%, 97%; *p* = 0.01) in the fish oil compared with the olive oil group.
Bisgaard 2011 [13]	736 pregnant women, unselected cohort	Pregnancy	Fish oil (2.4 g LCPUFA) vs. olive oil (placebo), daily from 24 weeks of gestation until 1 week after delivery	Persistent wheeze or asthma from birth to 3–5 years of age (primary outcome). Lower respiratory tract infections, asthma exacerbations, eczema, and allergic sensitization (secondary outcome).	Reduced risk of persistent wheeze or asthma (16.9% vs. 23.7%; hazard ratio, 0.69; 95% CI, 0.49, 0.97; *p* = 0.035). Reduced risk of lower respiratory tract infections (31.7% vs. 39.1%; hazard ratio, 0.75; 95% CI, 0.58 to 0.98; *p* = 0.033), but no effect on asthma exacerbations, eczema or allergic sensitization.
Noakes 2012 [14]	123 pregnant women, high-risk cohort for atopy	Pregnancy	Diet with 2 portions of salmon per week (providing 3.45 g EPA plus DHA) vs. habitual diet (which was low in oily fish), from 20 wk gestation until delivery	Clinical outcomes at 6 months (secondary outcomes)	No difference in the incidence of wheeze, eczema, lower respiratory tract infections, and allergic sensitization
Best 2018 [15]	701 pregnant women, high-risk cohort for atopy	Pregnancy	Fish oil capsules (900 mg of LCPUFA ~800 mg DHA and 100 mg EPA) vs. vegetable oil capsules without LCPUFA, daily from <21 weeks’ gestation until birth	Allergic disease symptoms (eczema, wheeze, rhinitis) at 1, 3, and 6 years of age reported by parents using a standardized questionnaire. Allergic sensitization assessed by skin prick testing.	No difference in wheeze symptoms with sensitization across the 1-, 3-, and 6-year assessments (adjusted relative risk 0.81, 95% CI 0.55, 1.21, *p* = 0.31)
Furuhjelm 2011 [16]	145 pregnant women, high-risk cohort for atopy	Pregnancy and Lactation	LCPUFA (1.6 g EPA and 1.1 g DHA) vs. placebo, daily from 25 weeks of gestation continuing through 3.5 months of breastfeeding.	Allergic disease in infants up to 2 years of age	No difference in cumulative and point prevalence at 2 years of age of asthma and allergic asthma, despite lower cumulative incidence of allergic sensitization and IgE-related disease up to 24 months of age (adjusted odds ratio 0.29, 95% CI 0.1–0.86. *p* = 0.03).

DHA, (omega-3 fatty acid) docosahexaenoic acid; EPA, (omega-3 fatty acid) eicosapentaenoic acid; IU, International Units; LC, long-chain; PUFA, polyunsaturated fatty acids; VD, vitamin D.

**Table 2:** Randomized controlled trials on vitamin D or omega-3 fatty acid supplementation during infancy for the primary prevention of pediatric wheeze/asthma.

Authors, Years	Population (*N*), Characteristics	Time of Exposure	Interventions	Outcomes	Findings on Wheeze/Asthma
Rueter 2020 [17]	195 infants, high risk for atopy, sufficient vitamin D levels at birth	Postnatal	400 IU VD3 vs. placebo, daily for the first 6 months of life	Allergic disease at 1 and 2.5 years of age	No differences in incidence for wheeze or recurrent wheeze/asthma at either 1 year (relative risk 1.66, 95% CI 0.92, 3.01; *p* = 0.13) or at 2.5 years of age (relative risk 1.32, 95% CI 0.79, 2.23; *p* = 0.38)
Hibbs 2018 [18]	300, black premature infants (born at 28–36 weeks’ gestation)	Postnatal	400 IU VD3/d vs. placebo (diet-limited supplementation), daily from birth to 6 months of life	Recurrent wheezing by 12 months’ adjusted age	Reduced risk of recurrent wheezing (31.1% vs. 41.8%; risk difference, -10.7%, 95% CI, -27.4%, -2.9%; relative risk 0.66, 95% CI, 0.47, 0.94; *p* =0.02)
Grant 2016 [19]	260, pregnant women and their infants	Pre and postnatal	Woman–Infant pair assigned to: placebo-placebo vs. 1.000 IU—400 IU VD3/daily vs. 2.000 IU—800 IU VD3 daily, from 27 weeks gestation to birth, and then to infants for the first 6 months of life	Aeroallergen sensitization and healthcare visit for acute respiratory illness (i.e., cold, otitis media, an upper respiratory infection, croup, asthma, bronchitis, bronchiolitis, a wheezy lower respiratory infection or fever and cough) at 18 months old	Differences in the proportion of children with primary care visits described by the doctor as being for asthma (11%, 0%, 4%, *p* = 0.02), but not for the other respiratory diagnoses
Mihrshahi 2004 [20]	376 infants, high risk for atopy	Postnatal	Tuna fish oil and omega-3-rich margarine and cooking oils vs. placebo (polyunsaturated margarine and cooking oils), from 6 months of life (or at the start of formula feeding)	Allergic sensitization and asthma/wheezing at 18 months old	Wheeze ever, doctor visits for wheeze, bronchodilator use and nocturnal coughing were significantly reduced in children in the higher quintiles of omega-3 fatty acid concentration in plasma (*p* < 0.05). No difference in diagnosed asthma or atopy between the exposure quintiles.
Marks 2006 [21]	516 children, high-risk for atopy	Postnatal	House dust mite avoidance (mattress cover) vs. placebo; dietary fatty acid modification (see reference ^95^) vs. placebo	Asthma, allergic sensitization, and eczema at 5 years of age	The prevalence of asthma, wheezing, eczema, or allergic sensitization did not differ between the diet groups (*p* > 0.1).
Foiles 2016 [22]	91 children	Postnatal	As infants, they were fed either a control formula without LCPUFA or one of three formulas that contained 0.64% of total fatty acids as arachidonic acid and either 0.32, 0.64, or 0.96% of total fatty acids as DHA	Allergic skin and respiratory illnesses through 4 years of age	If the mother reported allergy, the LCPUFA group had a 74% reduction (hazard ratio = 0.26; 95% CI 0.07, 0.9; *p* =0.02) in wheezing/asthma in the first 4 years of life compared to the control group, whereas LCPUFA and control groups did not differ if the mother reported no history of allergy (hazard ratio = 0.78; 95% CI 0.2, 2.9; *p* = 0.71)
Birch 2010 [23]	89 infants	Postnatal	DHA/arachidonic acid-supplemented milk formula (0.32%–0.36%/0.64%–0.72% of total fatty acids, respectively) vs. non-supplemented formula (control), fed during the first year of life	Upper respiratory infection (URI), wheezing, asthma, bronchiolitis, bronchitis, allergic rhinitis, allergic conjunctivitis, otitis media, sinusitis, atopic dermatitis (AD), and urticaria up to 3 years of age	Lower odds for developing URI (odds ratio 0.22, 95% CI 0.08, 0.58), wheezing/asthma (odds ratio 0.32, 95% CI 0.11,0.97) in the intervention group compared to controls.
D’Vaz 2012 [24]	420 infants, high-risk for atopy	Postnatal	Fish oil (280 mg DHA and 110 mg EPA) vs. placebo (olive oil), from birth to age 6 months	Eczema, food allergy, and asthma at 1 year of age	No significant differences in recurrent wheeze or persistent coughing between 6 or 12 months, but plasma DHA levels at 6 months significantly associated with less recurrent wheezing in the first year of life (*p* = 0.029)

Cfu, colony-forming units; DHA, (omega-3 fatty acid) docosahexaenoic acid; EPA, (omega-3 fatty acid) eicosapentaenoic acid; IU, International Units; lcFOS, long-chain fructo-oligosaccharides; LC, long-chain; PUFA, polyunsaturated fatty acids; pAOS, pectin-derived acidic oligosaccharides; scGOS, short-chain galacto-oligosaccharides; VD, vitamin D.

## Abbreviations

AGEs	advanced glycation end products
eHF	extensively hydrolyzed milk formulas
GINA	Global Initiative for Asthma Guidelines
RCTs	randomized controlled trials
LCPUFAs	long-chain polyunsaturated fatty acids
pHF	partially hydrolyzed formulas
VD	vitamin D
